# Impact of Solid State Roadway Lighting on Melatonin in Humans

**DOI:** 10.3390/clockssleep4040049

**Published:** 2022-11-18

**Authors:** Ronald B. Gibbons, Rajaram Bhagavathula, Benjamin Warfield, George C. Brainard, John P. Hanifin

**Affiliations:** 1 Virginia Tech Transportation Institute, Virginia Tech, Blacksburg, VA 24061, USA; 2 Department of Neurology, Thomas Jefferson University, Philadelphia, PA 19107, USA

**Keywords:** spectrum, melatonin, roadway lighting, light at night, LED, pedestrians, drivers, light trespass, road user health

## Abstract

Introduction: In 2009, the World Health Organization identified vehicle crashes, both injury-related and fatal, as a public health hazard. Roadway lighting has long been used to reduce crashes and improve the safety of all road users. Ocular light exposure at night can suppress melatonin levels in humans. At sufficient light levels, all visible light wavelengths can elicit this response, but melatonin suppression is maximally sensitive to visible short wavelength light. With the conversion of roadway lighting to solid state sources that have a greater short wavelength spectrum than traditional sources, there is a potential negative health impact through suppressed melatonin levels to roadway users and those living close to the roadway. This paper presents data on the impact of outdoor roadway lighting on salivary melatonin in three cohorts of participants: drivers, pedestrians, and those experiencing light trespass in their homes. Methods: In an outdoor naturalistic roadway environment, healthy participants (*N* = 29) each being assigned to a cohort of either pedestrian, driver, or light trespass experiment, were exposed to five different solid state light sources with differing spectral emissions and one no lighting condition. Salivary melatonin measurements were made under an average roadway luminance of 1.0 cd/m^2^ (IES RP-18 Roadway Lighting Requirements for expressway roads) with a corneal melanopic Equivalent Daylight Illuminances (EDI) ranging from 0.22 to 0.86 lux. Results: The results indicate that compared to the no roadway lighting condition, the roadway light source spectral content did not significantly impact salivary melatonin levels in the participants in any of the cohorts. Conclusions: These data show that recommended levels of street lighting for expressway roads do not elicit an acute suppression of salivary melatonin and suggest that the health benefit of roadway lighting for traffic safety is not compromised by an acute effect on salivary melatonin.

## 1. Introduction

In 2009, the World Health Organization identified vehicle crashes, both injury- related and fatal, as a public health hazard [1]. Roadway lighting has long been used as a tool to improve the safety of all road users including drivers and pedestrians and to reduce crashes [2]. Implementing light at night, particularly with newer technologies, can be problematic for the roadway users, those living close to the roadway, and to the general environment around the roadway.

Solid state lighting (SSL) has revolutionized street and area lighting. In the last decade, light-emitting diode (LED) sources have replaced high pressure sodium (HPS) sources for the purpose of illuminating roadways in the United States. A major difference in addition to light distribution and light output control is the spectral power distribution (SPD). The SPD of HPS and LED light sources differ greatly in terms of wavelength content but are often identified by the correlated color temperatures (CCT) measured in Kelvin (K). The CCT values are related in appearance to the absolute temperature of a black body radiator (incandescent source). This change in the SPD of the roadway lighting sources has sparked an intense debate over the effects of LED lighting on the health of road users and those adjacent to the roadway at night. The American Medical Association (AMA) has recommended the use of lower CCTs (3000 K or lower) to minimize the potential ill effects to human health (sleep disturbance and glare) and the environment [3].

The human body has 24-h circadian rhythms that are primarily driven by cycles of light and dark in the environment [4]. In addition, light has powerful acute neuroendocrine and neurobehavioral effects, including regulation of the hormone melatonin, which is secreted from the pineal gland. These effects are mediated primarily by light stimulation of the eye’s intrinsically photosensitive retinal ganglion cells (ipRGCs) [5]. For healthy circadian and neuroendocrine regulation, humans need exposure to sufficiently bright light during the daytime and darkness during the night. In modern, industrialized nations, concerns have been raised that lighting at night on roadways and outdoor spaces contributes to the disruption of normal sleep, circadian rhythms, and neuroendocrine physiology. Such disruption has been associated with risk of certain cancers, heart disease, and metabolic disorders [6,7]. The ipRGCs are most sensitive to blue light, with light in the range of 459–484 nm having the strongest impact on circadian, neuroendocrine, and neurobehavioral regulation. In outdoor environments, SSL primarily takes the form of LEDs for roadway lighting, which typically have higher short wavelength (blue) spectral content than traditional roadway lighting. Controlled laboratory studies have shown that exposures to LEDs in the evening and nighttime disturb circadian rhythms, suppress melatonin, and result in sleep loss [8,9,10].

While studies have shown the benefits of SSL in terms of energy consumption and visibility over traditional outdoor light sources such as HPS [11,12,13], the link between melatonin suppression and LED lighting at outdoor levels, however, has not been investigated in a natural environment. Currently, most of the existing data sets and literature are based on controlled laboratory experiments. This paper reports three experiments that tested the impact of roadway lighting on melatonin in vehicle drivers, pedestrians, and individuals in a bedroom close to the roadway. These experiments were performed in a naturalistic environment on a test track to simulate as close to a naturally occurring event as a possible.

The primary hypothesis tested in this research is that salivary melatonin levels will be suppressed due to the exposure to light from typical roadway lighting levels as experienced in separate cohorts of vehicle drivers, pedestrians and individuals experiencing light trespass in their homes. It is also hypothesized that light sources with higher blue content will suppress the salivary melatonin levels more than sources with lower blue content.

## 2. Results

### 2.1. Melatonin Assay Performance

The inter-assay Coefficient of Variation (CV) from the 19 salivary assays run for the exposure experiment ranged from 5.0% to 12.1%. The intra-assay CV calculated from three control samples at differing levels of melatonin assayed had a range of 2.4% to 6.9%. The minimum detection limit of the salivary assay was 1.0 ± 0.4 pg/mL melatonin.

For the control experiment, the inter-assay coefficients of variation (CVs) from the five plasma melatonin assays run for this study ranged from 2.9% to 14.0%. The intra-assay CV calculated from four control samples at differing levels of melatonin assayed had a range of 2.4% to 6.5%. The minimum detection limit of the plasma assay was 2.3 ± 0.8 pg/mL melatonin.

### 2.2. Control Results

#### 2.2.1. Drivers

**Figure 1:**
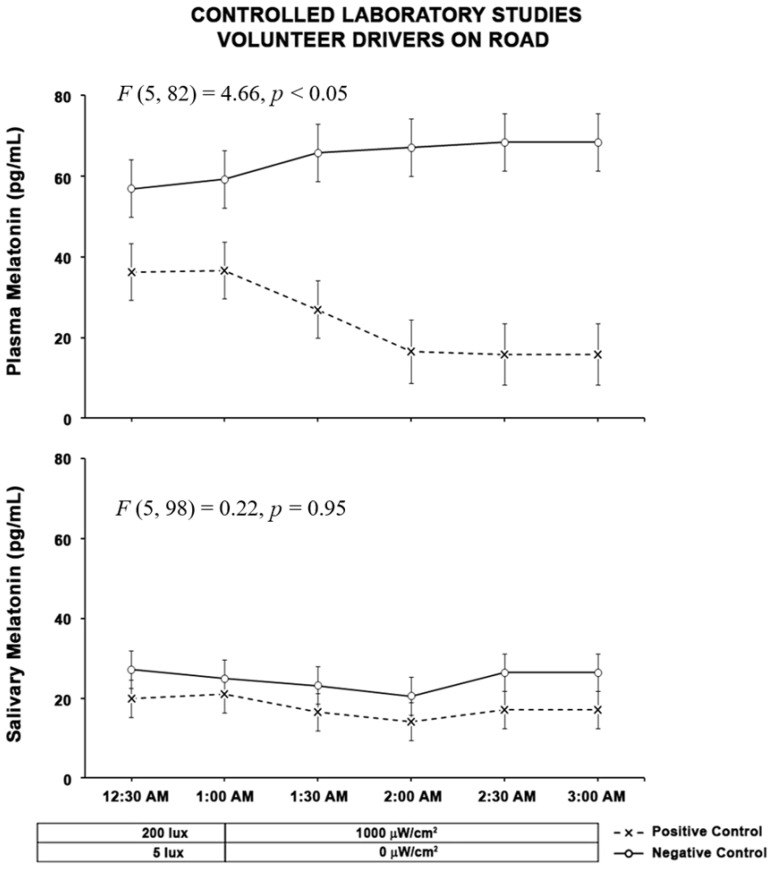
The two graphs above show results from subjects in the laboratory control studies. The “O” symbols represent the mean (± standard error of the mean, SEM) plasma and salivary melatonin levels during the negative control study when subjects were exposed to darkness from 1:00 A.M. to 3:00 A.M. The “X” symbols represent the mean (± SEM) plasma and salivary melatonin levels during exposure to 1000 µW/cm^2^ (2132 lx Melanopic Equivalent Daylight Illuminance (EDI)) electric light from 1:00 A.M. to 3:00 A.M. Statistical comparisons of melatonin values are for the exposure periods only (1:00 A.M. to 3:00 A.M.) and are based on Linear Mixed Model analysis (LMM). For the plasma melatonin suppression, the main effect of the type of test was significant (F (1, 12.2) = 34.42, *p* < 0.0001) and for the salivary melatonin suppression, the main effect of the type of test was significant (F (1, 17.2) = 8.16, *p* = 0.01).

**Figure 2:**
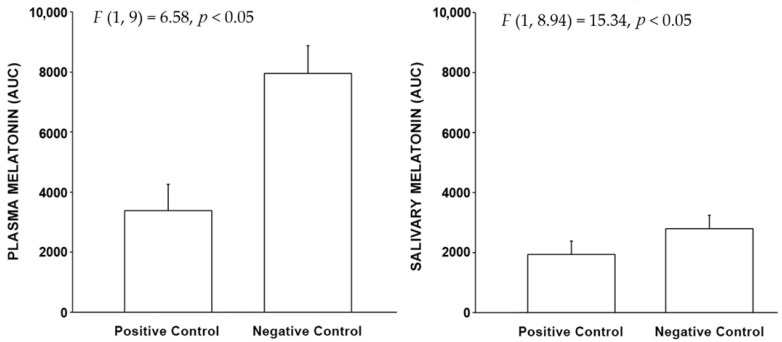
The bars shown above represent the mean (+ SEM) AUC plasma and salivary melatonin levels for the positive and negative control conditions. The integrated melatonin AUC values were calculated from the raw plasma and salivary melatonin values shown in Figure 1 for the exposure period only (1:00 A.M. to 3:00 A.M.). The statistics are based on the LMM.

For the drivers, the results of the positive and negative control experiments are shown in Figure 1 and Figure 2.

The results of a Linear Mixed Models Analysis (LMM) on the effects of the type of control on plasma melatonin suppression showed that the main effect of the type of test was significant (F (1, 12.2) = 34.42, *p* < 0.0001) and the main effect of time (F (4, 50.6) = 0.31, *p* = 0.87) or the interaction between type of test and time was not significant ((F (4, 59.1) = 1.29, *p* = 0.28). The results of the LMM on the effects of the type of control on salivary melatonin suppression showed that the main effect of the type of test was significant (F(1, 17.2) = 8.16, *p* = 0.01) and the main effect of time (F (4, 50.3) = 2.07, *p* = 0.10) or the interaction between type of test and time was not significant ((F (4, 56.2) = 0.20, *p* = 0.93).

The results indicate that the negative control results show the expected rise in both the plasma and salivary melatonin levels. It also shows in positive control results that the exposure from the test light suppressed the melatonin rise. The other critical aspect of the results is the comparison between salivary and plasma melatonin results. Here, the difference in the sensitivity to the melatonin level for the salivary measures are significantly lower than those for the plasma samples.

The area under the curve (AUC) Linear Mixed Model analysis also shows that the melatonin concentrations in the positive control were significantly lower than negative control experiments in both plasma and saliva (see Figure 2).

In both cases, the salivary sensitivity is lower than those for the plasma measures.

#### 2.2.2. Pedestrians

**Figure 3:**
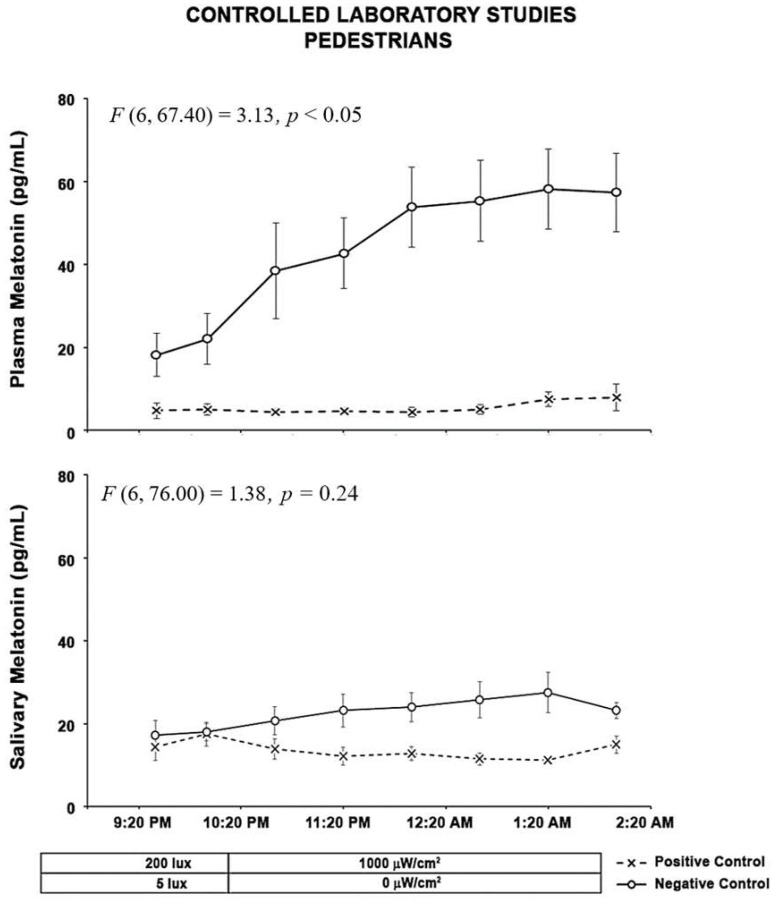
The two graphs above show results from subjects in the positive and negative control studies. The “O” symbols represent the mean (± standard error of the mean, SEM) plasma and salivary melatonin levels during the negative control study when subjects were exposed to darkness from 10:00 P.M. to 2:00 A.M. The “X” symbols represent the mean (±SEM) plasma and salivary melatonin levels during exposure to 1000 µW/cm^2^ (2132 lx Melanopic EDI) electric light from 10:00 P.M. to 2:00 A.M. Statistical comparisons of melatonin values are for the exposure periods only (10:00 P.M. to 2:00 A.M.) and are based on the linear mixed model (LMM). For plasma melatonin suppression, the results showed that the main effects of the type of test (F (1, 13.3) = 74.9, *p* < 0.0001) and time (F (6,68.2) = 11.75, *p* < 0.0001) were significant and the interaction between type of test and time was also significant ((F (6 ,67.4) = 3.13, *p* = 0.0091). For the salivary melatonin suppression, the main effect of the type of test was significant (F (1, 21.1) = 23.32, *p* < 0.0001).

**Figure 4:**
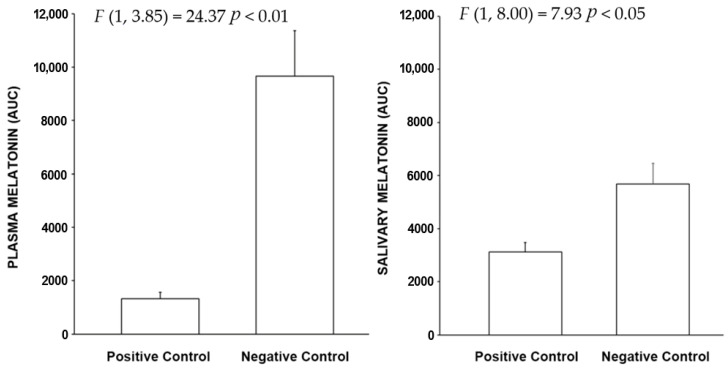
The bars shown above represent the mean (+ SEM) AUC plasma and salivary melatonin levels for the positive and negative control conditions. The integrated melatonin AUC values were calculated from the raw plasma and salivary melatonin values shown in Figure 3 for the exposure period only (10:00 P.M. to 2:00 A.M.) The statistics are based on the LMM.

For the pedestrian participants, the results of the positive and negative control experiments are shown in Figure 3 and Figure 4.

The results of the LMM analysis on the effects of the type of control on plasma melatonin suppression showed that the main effects of the type of test (F (1, 13.3) = 74.9, *p* < 0.0001) and time (F (6, 68.2) = 11.75, *p* < 0.0001) were significant and the interaction between type of test and time was also significant ((F (6, 67.4) = 3.13, *p* = 0.0091). The results of the LMM on the effects of the type of control on salivary melatonin suppression showed that the main effect of the type of test was significant (F (1, 21.1) = 23.32, *p* < 0.0001) and the main effect of time (F (6, 80.3) = 0.38, *p* = 0.89) or the interaction between type of test and time was not significant ((F (6, 76) = 1.38, *p* = 0.24).

The overall results for the control sessions are similar to those of the driver participants with a more pronounced rise in melatonin when compared to the driver participants. This is likely due to the timing of the experiment. The exposures for the pedestrian participants were started at 10 P.M. rather than 1 A.M. for the drivers.

The AUC results show a reduced sensitivity, however both measured methods show statistically significant differences in the measured effects.

#### 2.2.3. Light Trespass

The results of the LMM on the effects of the type of control on plasma melatonin suppression showed that the main effects of the type of test (F (1, 9.11) = 12.00, *p* = 0.0070) and time (F (4, 61.7) = 3.69, *p* = 0.0093) were significant and the interaction between type of test and time was not significant ((F (4, 70.3) = 1.10, *p* = 0.36). The results of the LMM on the effects of the type of control on salivary melatonin suppression showed that the main effect of the type of test was significant (F(1, 15.7) = 5.82, *p* = 0.03) and the main effect of time (F (4, 63.5) = 0.70, *p* = 0.60) or the interaction between type of test and time was not significant ((F (4, 66.5) = 1.24, *p* = 0.30).

**Figure 5:**
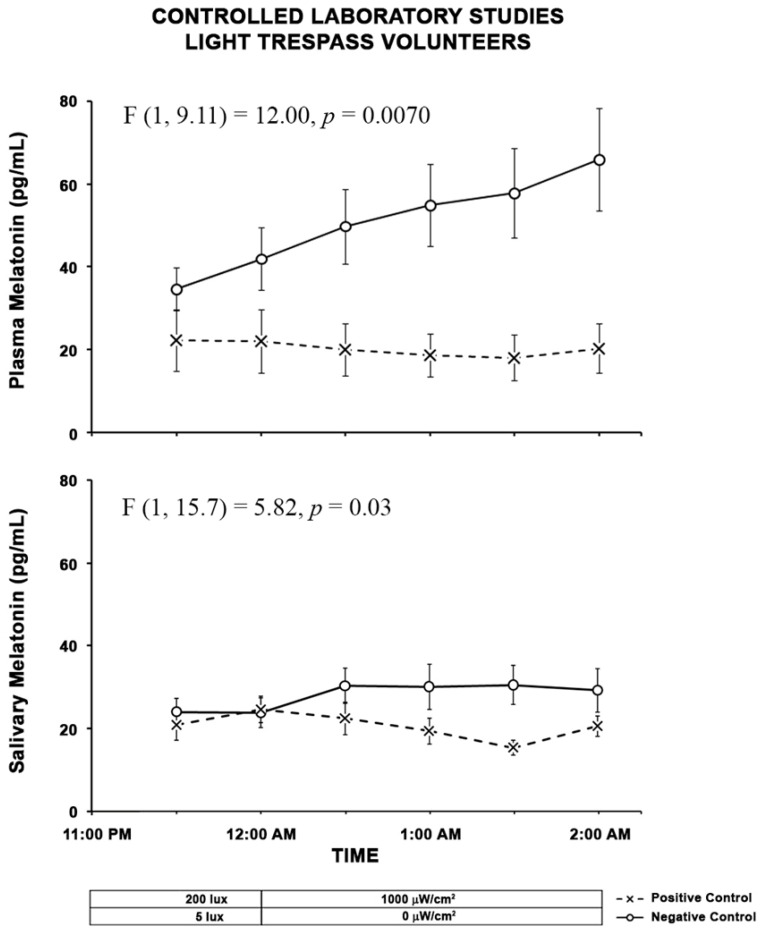
The two graphs above show results from subjects in the laboratory control studies. The O symbols represent the mean (± standard error of the mean, SEM) plasma and salivary melatonin levels during the negative control study when subjects were exposed to darkness from 12:00 A.M. to 2:00 A.M. The X symbols represent the mean (±SEM) plasma and salivary melatonin levels during exposure to 1000 µW/cm^2^ electric light from 12:00 a.m to 2:00 A.M. Statistical comparisons of melatonin values are for the exposure periods only (12:00 a.m to 2:00 A.M.) and are based on LMM. The main effects of the type of test (F (1, 9.11) = 12.00, *p* = 0.0070) and time (F (4,61.7) = 3.69, *p* = 0.0093) were significant for plasma melatonin suppression and for the salivary melatonin suppression, main effect of the type of test was significant (F(1, 15.7) = 5.82, *p* = 0.03).

For the light trespass participants, the negative control study shows a melatonin rise over the duration of the lighting exposure for both the plasma and the saliva measurements, which is similar to the pedestrian participants. Like the other data sets, the rise is clearly more pronounced in the plasma data (Figure 5).

**Figure 6:**
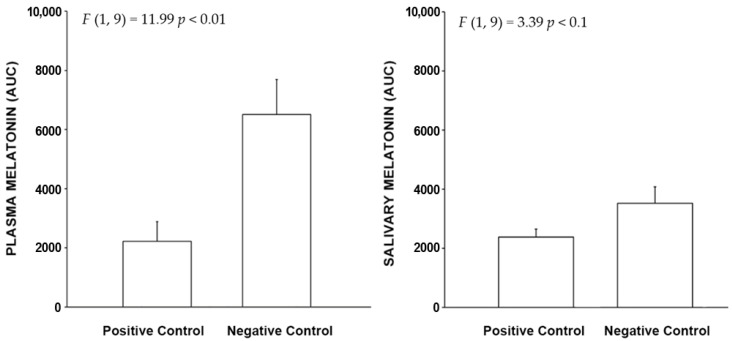
The bars shown above represent the mean (+ SEM) AUC plasma and salivary melatonin levels for the positive and negative control conditions in the laboratory portion of the light trespass study. The integrated melatonin AUC values were calculated from the plasma and salivary melatonin values shown in Figure 5 for the exposure period only (12:00 A.M. to–2:00 A.M.). The statistics are based on LMM.

The AUC results are shown in Figure 6. Like the time-based curves, the negative control levels are higher than the positive control levels, again demonstrating melatonin suppression due to bright light exposure in the positive control study. The differences in the AUC analysis were statistically significant for plasma (*p* < 0.01) and trended towards significance for salivary (*p* < 0.1) melatonin levels.

### 2.3. Exposure Results

#### 2.3.1. Drivers

**Figure 7:**
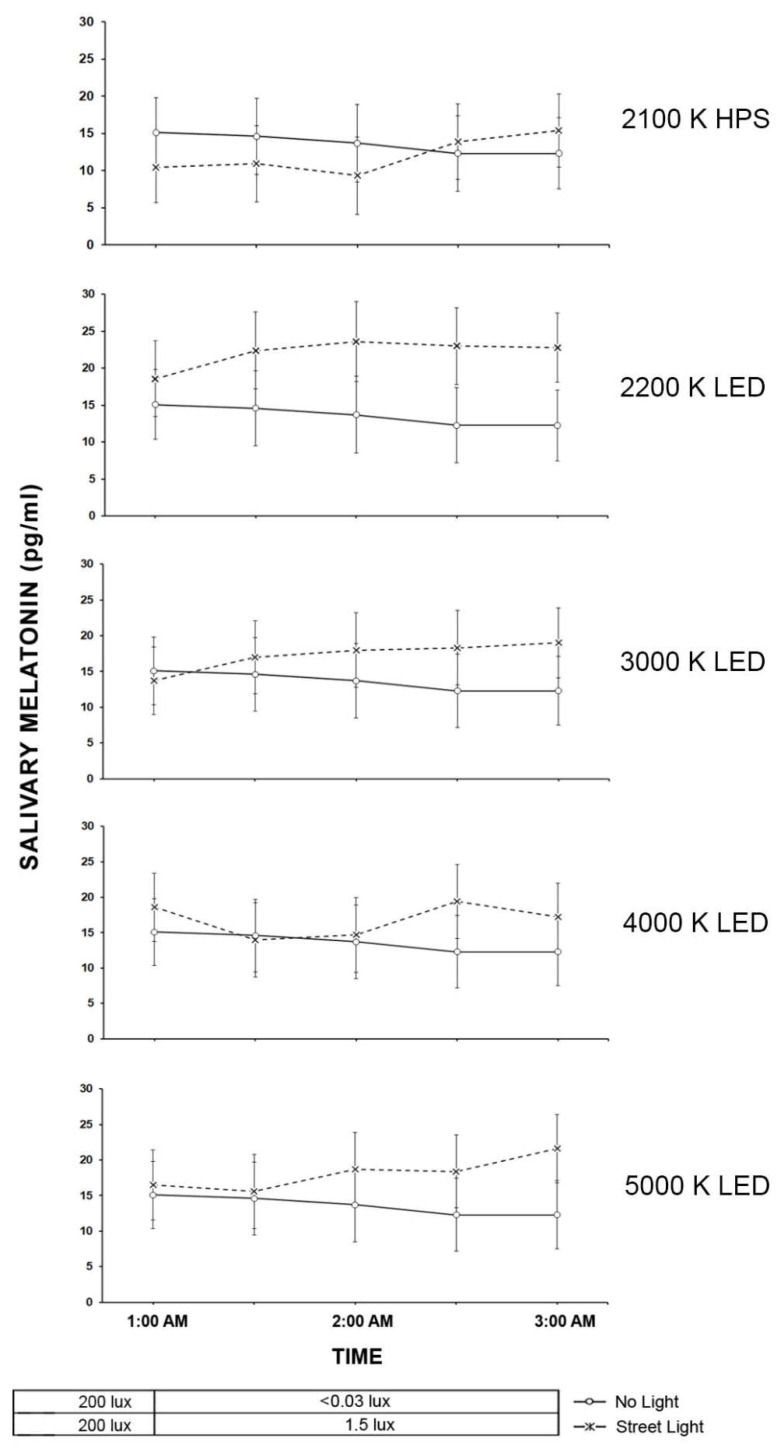
In the five graphs above, each of the roadway lighting conditions is compared to no roadway lighting for their effects on salivary melatonin of driver participants. The “O” symbols represent the mean salivary melatonin levels during the no roadway lighting condition (±SEM). The “X” symbols represent the mean salivary melatonin levels during exposure to roadway lighting (+SEM). The 1.0 cd/m^2^ luminance level (0.22–0.86 melanopic EDI) for all roadway lighting conditions from IESNA RP-18-14 (2014).

The results for the melatonin concentration measurements for the driver participant cohort based on experimental time are shown in Figure 7 for each of the tested roadway lighting configurations (dashed lines) as compared to the no roadway light condition (solid line). The error bars for each of the time-based measurements are the standard errors for each of the time-based measurements. The exposure times of the experiment are shown at the bottom of Figure 7.

**Figure 8:**
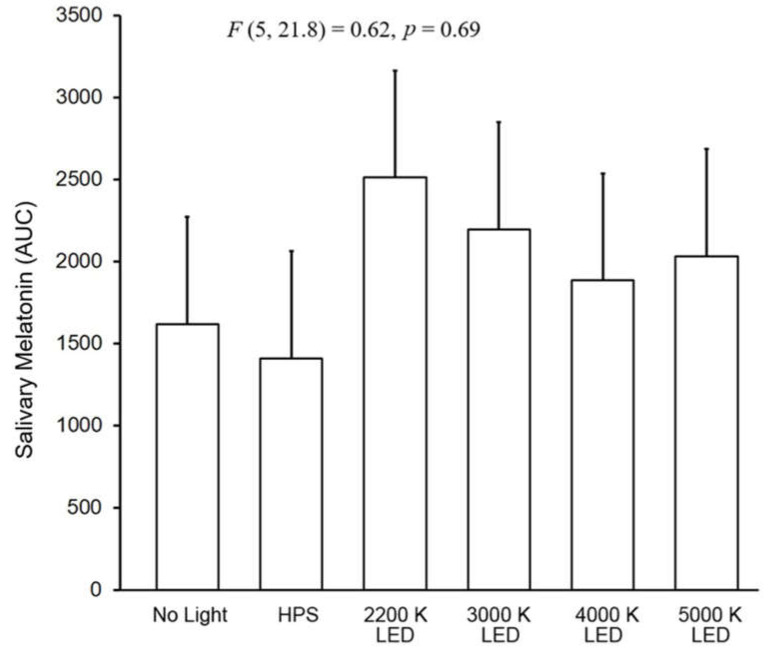
The bars shown above represent the AUC mean salivary melatonin levels (+SEM) for each of the six roadway lighting condition. The integrated melatonin AUC values were calculated from the raw salivary melatonin values shown in Figure 7 for the exposure period only (1:00 A.M.–3:00 A.M.). The statistics are based on the LMM.

In general, the driver participant results showed a rise in melatonin over time with no suppression based on the presence of roadway lighting. The results of the LMM on the effects of light source type on salivary melatonin suppression in the roadway lighting exposures showed the neither the main effects of light source type (F (5, 216) = 1.66, *p* = 0.15) and time (F (4, 213) = 1.13, *p* = 0.34) nor the interaction between them was significant (F (20, 224) = 0.68, *p* = 0.84). In Figure 8, the area under the curve calculation is shown which indicates the melatonin level over the course of the timed measurements. The results showed no statistical differences between any of the roadway lighting exposures including the no roadway lighting condition (F (5, 21.8) = 0.62, *p* = 0.69)

#### 2.3.2. Pedestrians

**Figure 9:**
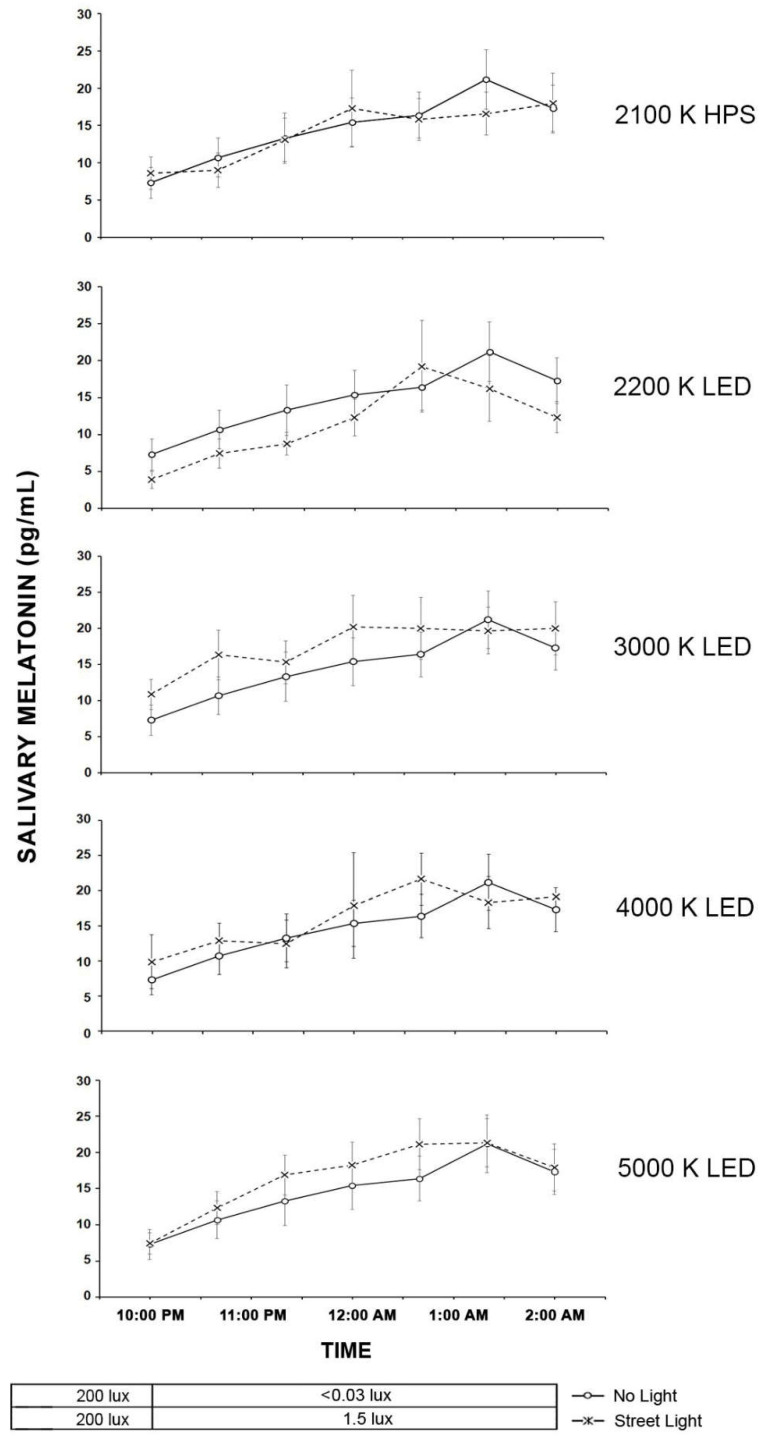
In the five graphs above, each of the roadway lighting conditions is compared to no roadway lighting for their effects on salivary melatonin of pedestrian participants. The “O” symbols represent the mean salivary melatonin levels during the no roadway lighting condition (±SEM). The “X” symbols represent the mean salivary melatonin levels during exposure to roadway lighting (+SEM). The 10 lx illuminance value (1.46–5.7 melanopic EDI) for pedestrian lighting conditions from IESNA RP-18-14 (2014).

Figure 9 shows the results for the pedestrian participants in the time exposure measurements. In general, the pedestrian participant results showed a rise in melatonin over time with no suppression based on the presence of roadway lighting. The results of the LMM on the effects of light source type on salivary melatonin suppression in the roadway lighting exposures showed that the main effects of light source type (F (5, 256) = 5.84, *p* < 0.00001) and time (F (6, 138) = 19.59, *p* < 0.0001) were significant but the interaction between them was not significant (F (30, 286) = 0.50, *p* = 0.99). Despite the significance of the main effect of light source, post hoc pairwise comparisons on the main effect of light source type revealed that there were no statistical differences in the salivary melatonin between the no roadway lighting condition and any of the roadway lighted conditions.

**Figure 10:**
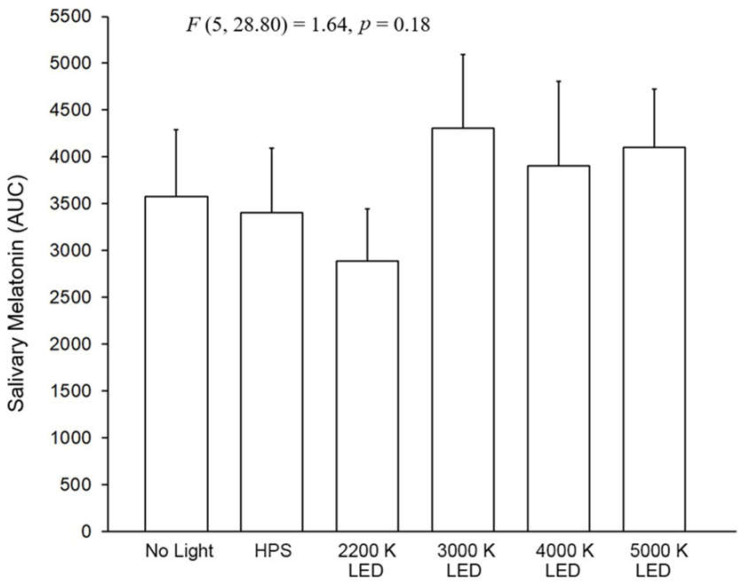
The bars shown above represent the AUC mean salivary melatonin levels in the pedestrian participants (+SEM) for each of the six roadway lighting conditions. The integrated melatonin AUC values were calculated from the raw salivary melatonin values shown in Figure 9 for the exposure period only (10:00 P.M.–2:00 A.M.). The statistics are based on the LMM.

Figure 10 shows the AUC results and the LMM statistics for pedestrian participants and each lighting exposure conditions. Here, the LMM shows no statistical difference (*F* (5, 28.80) = 1.64, *p* = 0.18) between the light exposure conditions.

### 2.4. Light Trespass

**Figure 11:**
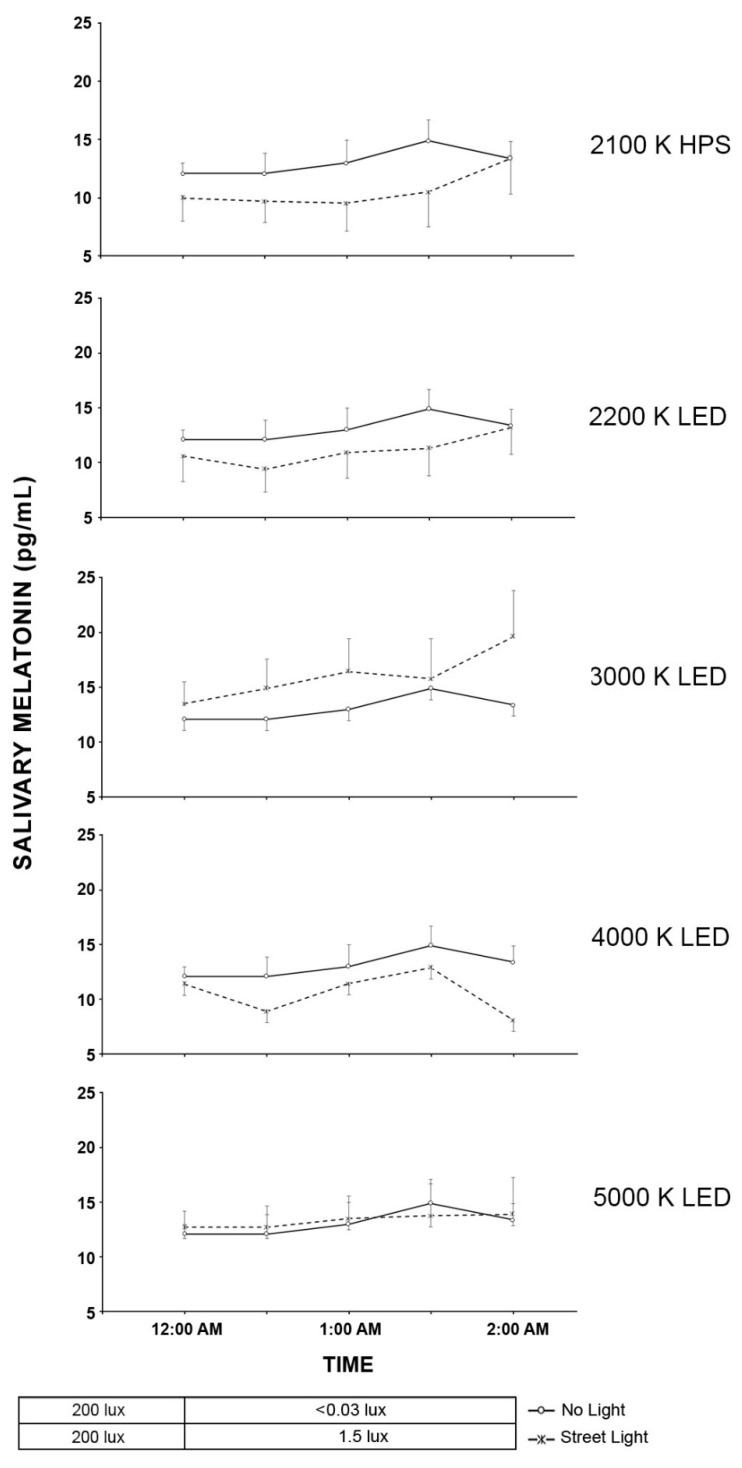
In the five graphs above, roadway light trespass from each of the street lighting conditions is compared to natural light trespass from the same street with no roadway lighting for their effects on salivary melatonin. The O symbols represent the mean salivary melatonin levels during the no electrical light condition (±SEM). The X symbols represent the mean salivary melatonin levels during exposure to roadway lighting (+SEM). The 1.5-lx illuminance value (0.22–0.86 melanopic EDI) for all roadway lighting conditions was based on the maximum allowable light trespass identified in IESNA RP-18-14 (2014).

**Figure 12:**
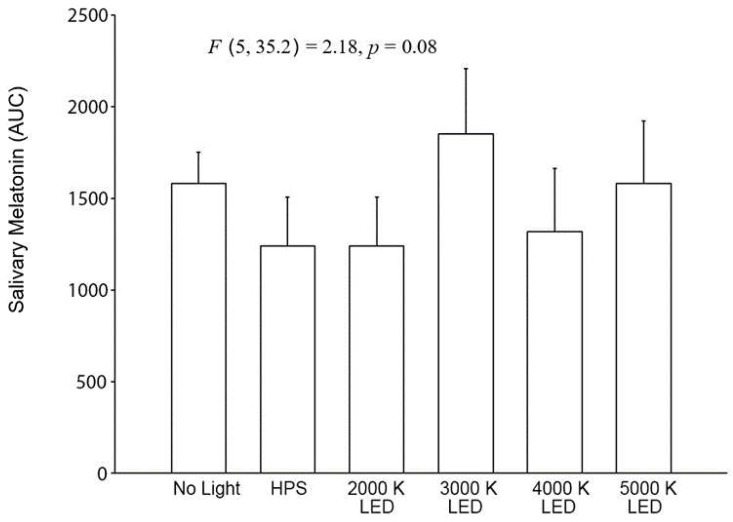
The bars shown above represent the AUC mean salivary melatonin levels (+SEM) for each of the six experimental conditions in the light trespass study. The integrated melatonin AUC values were calculated from the salivary melatonin values shown in Figure 11 for the exposure period only (12:00 A.M.–2:00 A.M.). The statistics are based on the LMM.

The results from the roadway lighting exposure experiment are shown in Figure 11 for the time-based results and in Figure 12 for the AUC calculations for the Light Trespass participants.

The results of the LMM on the effects of light source type on salivary melatonin suppression in the roadway lighting conditions showed that the main effects of light source type was significant (F (5, 160) = 5.51, *p* < 0.0001) but neither the main effect of time (F (3, 84.5) = 1.61, *p* = 0.20) nor the interaction between light source type and time was significant ((F (15, 173) = 0.51, *p* = 0.93). Similar to the pedestrian cohort, despite the significance of the light source main effect, post hoc pairwise comparisons revealed that, for each of the streetlight sources, no statistical differences were observed for the change in the melatonin level under the streetlight exposure compared to the no light roadway condition. Similarly, the AUC comparisons showed no statistical differences between the six roadway conditions (*F* (5, 35.2) = 2.18, *p* = 0.08).

## 3. Discussion

### 3.1. Control

The data from the control session highlight two aspects of the data collection protocol.

Firstly, from the control sessions, a rise in melatonin would be expected in each of the experimental types. The timing of the experiment, specifically in the pedestrian and trespass participants, was established which created a melatonin rise in the negative control and a suppression in the positive control conditions. This validates the experimental design used for the experimental sessions.

The second aspect is the control session show the reduced sensitivity in the salivary versus plasma melatonin data collection sampling methods. This reduced sensitivity is a consideration discussed below in study limitation.

### 3.2. Exposure

The goal of this study was to evaluate melatonin suppression due to exposure to typical roadway lighting levels as experienced by three separate groups of roadway users (drivers, pedestrians, and individuals in a bedroom experiencing light trespass). From the results of this study, as compared to the no roadway lighting condition, no statistical differences in salivary melatonin suppression were observed between any of the roadway lighting conditions of different SPDs inclusive of those with higher blue light content. This was evident in both the time based results and the total melatonin content AUC calculations.

It must be remembered that the impact of light on melatonin has four components to it: spectrum, lighting level, exposure duration, and timing of exposure. In each arm of this study, the spectrum was evaluated at recommended light levels for expressway roadway lighting [14], while the light luminance, timing (See Table 1), and duration were kept constant. It must also be noted that the results from this investigation are based on an approach to consider the impact of light on salivary melatonin levels in a naturalistic environment. The activities undertaken by the participants were realistic and context specific: the drivers were driving a real vehicle while performing an object detection task on a lighted roadway, the pedestrians were allowed to perform activities like reading and playing quiet games under roadway lighting, and the light trespass participants were laying in a bed in a bedroom adjacent to a lighted roadway and were able to close their eyes if desired. Consequently, the results from the experiment can be readily applied to comparable urban and suburban conditions. This shows that light exposure from any of the naturalistic roadway lighting conditions, at the highest level of 10 photopic lux (in the pedestrian cohort) is not strong enough to elicit a detectable salivary melatonin suppression response in healthy participants.

It should be cautioned that describing light solely with photopic illuminance (V_λ_) is inappropriate for characterizing light that elicits the neuroendocrine effect of melatonin suppression explored in this study. In spite of this caution, throughout this study, photopic lighting levels were used for characterizing the experimental conditions based on the Recommended Practice for Roadway Lighting RP-8 [14] that specifies roadway conditions using photopic measurements. All five human photoreceptor types can contribute to melatonin regulation [5,15]. The recent CIE 2019 position statement, it is currently recommended to use alpha-opic melanopic equivalent daylight (D65) illuminance (EDI) for specific lighting designs and applications in typical everyday life for people with a regular, day-active schedule [16]. The melanopic EDI values reported in Table 2 for all conditions do not rise above 1 melanopic EDI. Indeed, the empirical melatonin results achieved in this trial are not surprising as this value has been recommended as a target to be achieved as a maximum ambient melanopic EDI for the sleeping environment in recommendations being put forth by the 2nd International Workshop on Circadian and Neurophysiological Photometry in 2019, which brought together experts in the physiological effects of lighting [17].

Notably, the IES recommends the highest street lighting luminance of 1.2 cd/m^2^ for a major roadway category with high pedestrian volume [14]. The tested street lighting conditions (1.0 cd/m^2^ based a on an expressway roadway category) were very close to this recommended luminance. With the results of the current study together with the recent international consensus [17], it could be hypothesized that a slightly higher value would not result in significant suppression of salivary melatonin. Importantly, in a recently published study, performed on the same test facility, a roadway lighting condition of 1.5 cd/m^2^ for one light source SPD was tested and no significant salivary melatonin suppression was observed ([18]).

There has been significant discussion on the selection of light sources used for roadway lighting design. There have been contentions that the light spectrum used in roadway lighting can suppress melatonin. It is clear that proper roadway lighting can contribute to safety of people and traffic [2,14]. A fundamental concept in modern medicine is that agents that can contribute to human health also have the potential capacity to cause harm. Disruption of melatonin regulation, circadian physiology and sleep by inappropriate light exposure has been linked to several diseases and disorders. As an example, epidemiological evidence reveals an association between breast and prostate cancer risk and shift work [7,19,20,21]. In the context of this emergent biomedical literature, the American Medical Association (AMA) released two public health reports on the effects of nighttime lighting on humans and the environment [22]. The more recent AMA report covers primarily nighttime LED outdoor community and street lighting as it pertains to visibility glare, visual impairment due to stray light, and the potential environmentally disruptive effects on humans [3]. This document provided guidance on the use of roadway lighting, indicating that 3000 K light sources should be the maximum used, as was estimated that a ‘white’ LED lamp is at least 5 times more powerful in influencing circadian physiology than a high pressure sodium light based on melatonin suppression” [3]. The 2016 AMA report prompted some response and controversy from the lighting and energy communities [23,24]. In terms of melatonin, it is noteworthy that the studies used for this recommendation are typically controlled laboratory studies or observational epidemiological studies. It is noteworthy that the AMA’s recommendation is based on a calculated estimate and not on actual measured data. As mentioned, the impact of a light source on melatonin is based on four components: light level, spectrum, timing and duration. Only spectrum was considered in the AMA recommendations. The data from this study indicate that the light source illuminance recommended in roadway applications is too low to impact salivary melatonin levels. Hence, when the light levels, duration and timing were held constant, the spectrum of the light source did not significantly impact acute salivary melatonin regulation. This indicates that the spectrum choice is not critical for a well-designed and well-controlled lighting system that meets the international criteria recommended for expressway roads lighting [14].

It is important to recognize that this study has some limitations. The Smart Road did not have any light sources in addition to the fixed overhead roadway lighting. Further, the light level selected for the roadway and for the trespass in the current study represented the higher end of light IES specifications [14]. In a typical lighted roadway environment, however, additional sources of illumination such as stray light from commercial establishments, apartment buildings, parking facilities, vehicle headlamps and the like, could potentially increase the amount of light a person might experience. The use of only fixed-overhead lighting in the current study helped isolate the effects of roadway lighting on melatonin suppression. These results are applicable to areas with no additional outdoor light sources other than roadway lighting, such as rural areas and suburban streets. Future research should continue to evaluate melatonin suppression in realistic urban and suburban environments.

Another limitation is that compared to quantification of plasma melatonin, the measurement of salivary melatonin has lower sensitivity for detecting changes in melatonin levels [25]. The results of the control experiments with plasma and salivary melatonin show that in terms of AUC, the salivary measurement was sufficiently sensitive for detecting melatonin changes in the control laboratory scenarios. It is possible the quantification of salivary melatonin might not have been sensitive enough for subtle melatonin changes in the naturalistic roadway studies.

A further limitation is the choice of a 200 lux conditioning room exposure to partially control for previous light exposure history. It was decided that a more naturalistic pre-test condition would be from an illuminated indoor environment so a 200 lux level was selected to represent an office or residential space exposure level (based on IES RP-11, 2020 [26] recommended levels for home office applications) This may have impacted the results as the majority of controlled laboratory studies on the neurophysiological effects of light are set against a dim or dark background illuminance precondition.

Another limitation to consider is that the impact of light on melatonin is not the sole indicator of human health. Measurement of longer-term effects on physiological and behavioral parameters like sleep efficiency, total sleep time, sleep onset, number of awakenings, and the like may show different impacts of roadway lighting.

Roadway lighting reduces vehicle crashes and fatalities resulting in improved public safety and health. Recommended expressway lighting levels appear to have minimal impact on salivary melatonin levels in humans suggesting minimal negative impact on this aspect of health. There are some caveats to this discussion, however, in that the measurements of the impact on melatonin and safety are only assessed at recommended lighting levels: over-lighting, badly controlled lighting installations, and wasted uplight can still be problematic for all human users and the environment as well as flora and fauna.

## 4. Methods

This study was approved by the Virginia Tech Institutional Review Board (IRB) and study procedures conformed to Virginia Tech safety regulations (IRB Reference Number 18–267 approved 4 May 2018). Each of the three roadway lighting exposure experiments was conducted at the Virginia Tech Transportation Institute (VTTI) and utilized test areas in the facility and the variable lighting system on the Virginia Smart Road.

The experimental sessions included indoor control exposure sessions that measured participants’ plasma and saliva melatonin concentrations, and on-road lighting exposure sessions where participants’ salivary melatonin concentrations were measured. The control sessions ensured that the participants demonstrated a normal melatonin rise in darkness, suppressed melatonin levels with a bright light exposure and was used to verify the link to the saliva measures.

### 4.1. Control Experiment Methods

A control protocol was performed to validate the experimental protocol in terms of the both the possibly of initiating a normal evening rise in melatonin levels in the participants as well as evaluating the impact of using salivary melatonin samples versus plasma melatonin samples.

The control protocol consisted of both a positive control where the participant was exposed to a lighted test condition where a melatonin suppression was expected and a negative control where the participant was in a dark condition expected to allow a normal rise in melatonin.

Each participant experienced the control conditions in the two weeks prior to the experimental sessions. The control sessions took place in a test area outfitted with booths with an exposure lighting system. The timings of the exposure match those experienced by the participants in the experimental sessions.

#### 4.1.1. Control Exposure Space

Test rooms were set up for positive and negative control light exposures. This space allowed six participants to be exposed individually using light exposure panels. A comfortable chair was provided for each participant to ensure that the participant remained seated upright during the exposure period. Each exposure station was optically isolated from the others so that positive and negative control tests could be performed simultaneously.

For the positive control, a light panel was built to provide uniform lighting to the participant’s eyes and to fill the retinal field with lighting of 1000 µW/cm^2^ measured at measured at eye level of the seated participant (~1.2 m from the floor; 2132 lx melanopic EDI). The panels measured 0.61 × 1.22 m (2 × 4 ft.) and housed commercially available 4000-K LED light sources with the SPD shown in the primary paper. The panels were driven by a dimmable electronic driver to provide stabilized output control of the fixture.

#### 4.1.2. Positive and Negative Control Sessions

Positive and negative control exposure sessions were used to establish each participant’s baseline suppressed and normal melatonin levels. Blood and saliva samples were drawn from the participants during these control sessions. In the positive control session, high nocturnal melatonin secretion was expected to be strongly suppressed. In the dark exposure negative control session, the participant was expected to exhibit the normal evening onset of melatonin production and higher melatonin secretion during the first half of the night.

For both control sessions, participants were seated in exposure booths following the conditioning period. During the positive control, participants were instructed to look toward the bright white light panel (1000 µW/cm^2^, 2132 lx melanopic EDI) for the duration of the session. During the negative control session, participants sat in the same booths, wore a black sleep mask, and had no light exposure.

During both control sessions, samples of blood were collected every 30 min through an indwelling intravenous catheter located in a forearm vein into 3-mL polystyrene tubes which contained 5.4 mg of K2 ethylenediaminetetraacetic acid (EDTA) for the measurement of plasma melatonin (BD Diagnostics, Franklin Lakes, NJ, USA).

Additionally, two saliva samples (2 mL) were collected with “Salivette” collection tubes every 30 min (Sarstedt, Inc., Hayward, CA, USA). Any drink or food, with the exception of water, was denied to the subject for 30 min prior to sampling. The swab was left in the mouth for 2–4 min while the subject gently chewed on it to stimulate saliva flow.

### 4.2. Exposure Experimental Design

In this study, three experiments were conducted based on the type of exposure; drivers, pedestrians, and individuals in a bedroom close to the roadway which will be referred to as light trespass going forward. For each of the three experiments, the exposure condition varied in duration, timing, and sampling rate (see Table 1). Note that a 2-h lighting conditioning session was used before each on-road experimental exposure session which began at the start time indicated. Roadway luminance was consistent across the three studies.

The dependent variable was the concentration of melatonin in saliva in picograms per milliliter (pg/mL) measured at set time intervals. While it was desirable to collect plasma samples at the outdoor test facility, there was no way to safely draw blood and comply with IRB and safety regulations. Consequently, for the on-road lighting exposures, only salivary samples were collected.

The independent variables for each of the experiments was lighting condition with each participant experiencing 5 different lighting spectral compositions (2100 K HPS, 2200 K LED, 3000 K LED, 4000 K LED and 5000 K LED) and a no roadway lighting condition. Lighting conditions were counterbalanced across each of the participants.

### 4.3. Equipment and Materials

#### 4.3.1. Light Conditioning Space

To partially control for previous light history, which is known to affect melatonin levels [27,28], a light conditioning space was used at the beginning of each experimental session. The in-building conditioning space was set at 200 lx (87.09 melanopic EDI) measured at the eye level of the seated participant (~1.2 m from the floor). The SPD of the light source used in the conditioning space (4000 K booth shown later in this paper).

#### 4.3.2. Smart Road Facility

The outdoor exposure scenarios were performed on the Virginia Smart Road, which is a 2.2-mile controlled test track located adjacent to VTTI. The capabilities of the test track allowed for variable lighting and the drivers exposure whereas temporary facilities were established for the light trespass and the pedestrian exposures. Each lighting tower on the Smart Road is equipped with 3 luminaires to facilitate the counterbalancing of the light source presentation.

**Figure 13:**
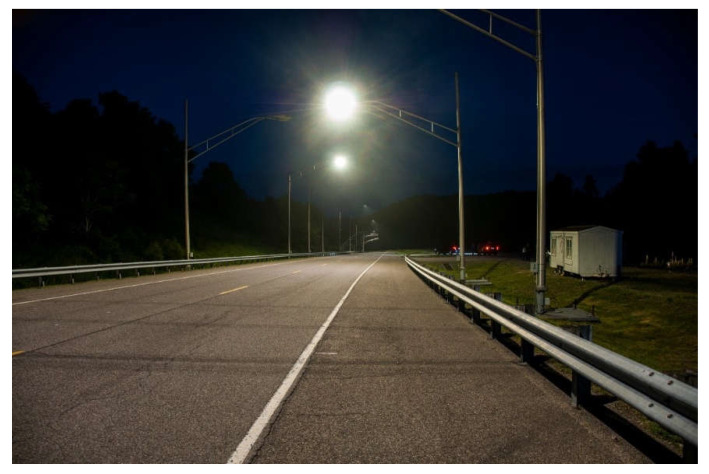
Smart Road Driving Facility.

For the driving experiment, the test road was used as a driving track where vehicles were able to travel through the lighted area. The vehicles were able to turn around at each end of the lighted area to ensure that the driver was exposed to the lighting condition at all times (Figure 13). The vehicle headlamps were also used at all times. The light exposure at the drivers’ eye was 1.4 lux (0.6 lux melanopic EDI).

**Figure 14:**
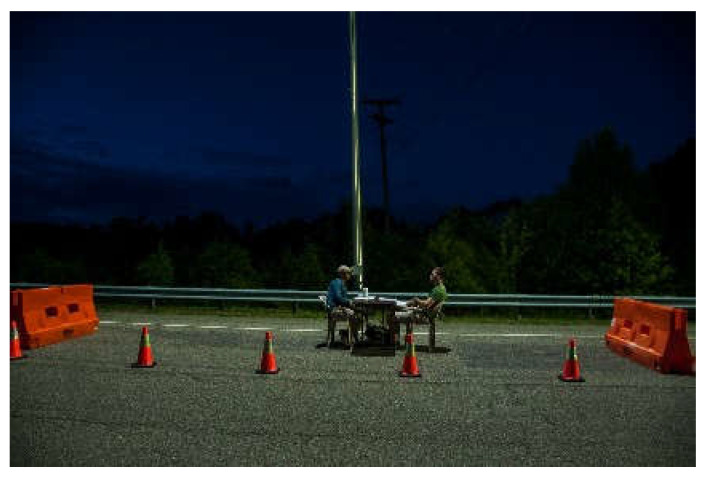
Pedestrian Test Location.

For the pedestrian experiment, a portion of the Smart Road was blocked off by barriers in a space away from the driving area so no vehicles were present. A table and chairs were located for the comfort of the participant (Figure 14). The chairs were located such that the exposure at the eye of the participant (1 m from the ground) was 10 vertical lux (4.3 lux melanopic EDI).

**Figure 15:**
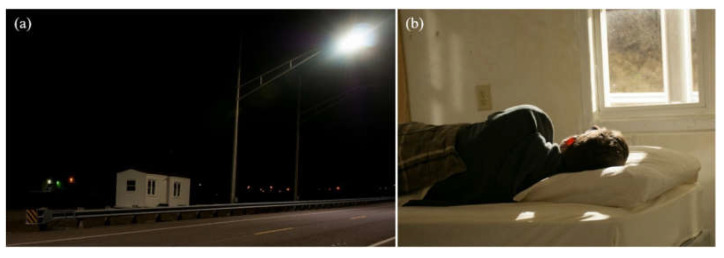
(**a**) Location of the simulated bedroom inside a trailer. (**b**) Participant experiencing the light trespass in the simulated bedroom.

In the light trespass experiment, a sleeping space was constructed in a small structure divided into matching 10 ft × 10 ft rooms, each with a 3 ft × 4 ft uncurtained window, a door, and a twin-sized bed. This structure was located on the side of the roadway, laterally 3 m (9.8 ft) from the luminaire pole and 8 m (26.2 ft) from the luminaire itself (Figure 15). The position of the sleeping rooms with respect to the luminaire was designed such that the lighting level on the pillow was 1.5 lx (0.22–0.86 melanopic EDI, see Table 2) in each room.

For the lighting used in the experiments, the Smart Road facility’s variable lighting system was configured with twelve paired lighting poles with 80-m spacing, for a total length of 960 m. Each pole is outfitted with a height-adjustable, triple tenon arm that enables three luminaires to be mounted per light pole. This allowed for all five lighting systems used in this experiment to be mounted simultaneously, facilitating the counterbalancing of participant exposures. All the luminaires were controlled by a wireless, independently addressable control system, which allowed the lighting level of the luminaires to be matched across each of the lighting systems tested.

The lighting configurations were established using five different commercially available luminaires, including a 400-W HPS cobra head at 2100 K; 2200 K LED luminaires with light guide optics; and three sets of matched LED luminaires with 3000 K, 4000 K, and 5000 K optic arrays. The LED luminaires had a Type II, medium roadway optical pattern.

**Figure 16:**
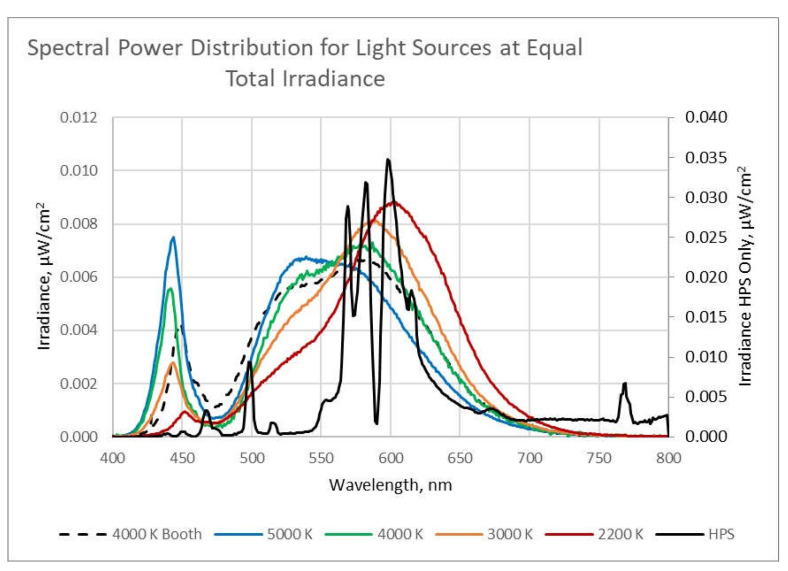
SPDs of the light sources used in the experiment. All light sources are plotted relative to the left-hand vertical scale, except for the HPS irradiance, which is plotted versus the right-hand vertical scale.

The SPDs from the roadway luminaires and the 4000 K LED exposure light utilized for conditioning and the positive control are shown in Figure 16. In the figure, the light spectra are plotted as irradiance versus wavelength for equal total irradiance. Since total irradiance is equal to the area under the curve (AUC), the HPS peak irradiance is much higher than the LED peaks and therefore is plotted on a secondary *y*-axis on the right of the chart.

In addition to the CCT and the photopic illuminance, the relative alpha-opic flux for all the luminaires versus the five major photoreceptor types found in the human eye were considered in this assessment: cyanopic, chloropic, and erythropic correspond to the short wavelength (blue), middle wavelength (green), and long wavelength (red) cones, respectively. Rhodopic is related to the sensitivity of the rods, and melanopic refers to the ipRGC melatonin response. These values are expressed in EDI, which is the metric accepted by the International Commission on Illumination (CIE), and defines the amount of daylight exposure required to elicit the same melanopsin response as the source being evaluated [15,29]. As shown in Table 2 these alpha-opic lux values are relative to a 1.5-lx photopic illuminance from each luminaire and represent the exposure experienced by the participants in the test space.

**Figure 17:**
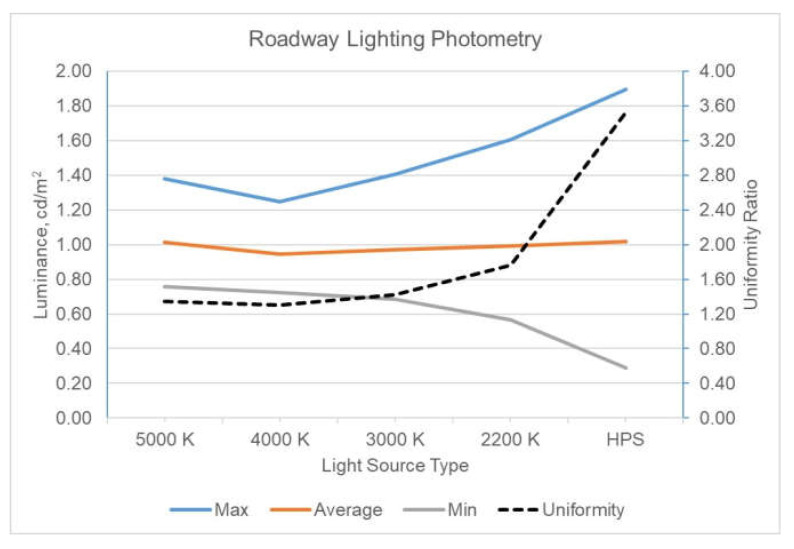
Roadway photometry by luminaire.

After installation, photometry of the luminaires was completed using a calibrated photometer (ProMetric PM-9913E-1, Radiant Imaging^®^, Redmond, WA, USA). The luminaires were photometrically characterized and dimmed to provide 1.0 cd/m^2^ average luminance (the calibrated photometer provided an average luminance when a polygon was drawn on the area of the road surface between two luminaire poles 80 m apart) on the roadway aligning with the IES RP-18 Roadway Lighting Requirements for the expressway road category. Figure 17 shows the average luminance of 1.0 cd/m^2^ across all lighting configurations. The uniformity of the light level on the roadway increased between the LED and HPS, which is typical of the differences between LED and HPS light sources.

### 4.4. Procedure

Participants were recruited using the VTTI participant database, online advertisement, and word of mouth. The first point of contact for recruitment was to administer a phone screening to determine eligibility for the study.

After recruitment of the participants for the experiment, each participant was assigned to a specific experiment type: drivers, pedestrians, or light trespass. Experimental sessions for each participant consisted of an orientation session, two control exposure sessions, and six road lighting exposure sessions each separated by at least one week.

Participants who completed any portion of the experiment were paid $25 per hour for their time. Data collection began on 8 July 2018 and was completed on 4 December 2018.

#### 4.4.1. Orientation Session

During the orientation session, participants read and signed the informed consent form, and completed vision tests. Participants were required to have a binocular visual acuity of 20/40 or better (measured by an Early Treatment Diabetic Retinopathy Study chart with an illuminator cabinet), and normal color vision (measured by Ishihara Color Vision test). Each participant was briefed and provided with an actigraphy monitor (ActiGraph wGT3X-BT, Actigraph, Pensacola, FL, USA) and a sleep log. Previous studies [30,31] have confirmed the validity and reliability of the model of the actigraphy monitor used in this study to estimate sleep/wake cycles. Participants were instructed to wear the actigraphy monitor for the duration of the experiment.

Each participant was instructed to adhere to a list of study constraints designed to stabilize their melatonin rhythm. These restrictions included elements such as limiting caffeine intake, regulating sleep/wake times, and limiting travel to within one time-zone. Participants were instructed to keep a consistent bedtime no later than midnight for the duration of the study and track the times they went to bed and woke up each day in the sleep log. Participants’ first experimental session was scheduled at least 2 weeks after their orientation so that their sleep activity could be verified using the actigraphy data and sleep logs.

#### 4.4.2. Experimental Exposure Sessions

Each of the six road lighting exposure sessions included an indoor conditioning period followed by the roadway exposure period. For all exposure sessions, participants were picked up at their homes and driven to the research facility by staff members.

#### 4.4.3. Conditioning

For each session, participants surrendered their cell phones and electronic devices. Their actigraphy data and sleep logs were reviewed for compliance. They were then escorted to the light conditioning room. and stayed there for 2 h until the road lighting exposure began and were permitted to engage in quiet activities such as reading or playing board games. For all exposure sessions, the light level was 200 lux (87.09 Melanopic EDI,) measured vertically at the eye.

#### 4.4.4. Road Lighting Exposure Sessions

After the completion of the positive and negative control sessions (discussed in the Supplementary Materials), the on-road experimental exposure sessions began. Each participant experienced exposure to six different road lighting conditions, with each session separated by a week.

For each test session, when the indoor conditioning period was complete, participants were escorted outside to a waiting vehicle. During this transition, the lighting in the hallways of the facility and the vehicle interior lights were dimmed (0.15 lx, 0.14 lx melanopic EDI). An experimenter drove the participants onto the Smart Road and escorted participants to the test location based on the experiment type.

For the drivers, they were escorted to the experimental vehicles (two matched 2017 Ford Explorers). Here, the participant sat in the driver seat and was oriented to the vehicle. They were then told the route of the vehicle which looped within the lighted area of the Smart Road. They were also instructed regarding a roadside visual detection task they were to perform while driving. Once oriented to the experiment, the driver then proceeded to drive the loops on road at 35 mi/h (56 km/h) stopping every 30 min where 2 saliva samples were collected. As the participants were tested in pairs, care was taken to ensure that the drivers did not pass each other where they would be exposed to the other vehicle headlamps.

The pedestrians were instructed to sit at a table on the secure portion of the roadway. The pedestrians were allowed to read, talk and play games. Pedestrians were also allowed to stand and walk but vigorous activities were not allowed. Participants also performed a short gap acceptance task where the participant stood on the shoulder of the road, an experimental vehicle driven by an experimenter approached and the participant declared if they would be willing to cross the road. Two saliva samples were collected as described above every 40 min for the duration of the session.

The light trespass participants were taken to the simulated bedroom. The participants each entered one of the bedrooms, where they were tasked with lying in the bed for two full hours of exposure time. Participants were instructed to lie facing the window and were allowed to close their eyes or sleep. Saliva samples were collected as described above every 30 min for the duration of the session.

At the completion of the experimental session, the participants’ belongings and actigraphy monitors were returned and they were driven home by lab staff.

#### 4.4.5. Sample Processing

The plasma and saliva samples were processed in the VTTI laboratory for melatonin assay. The saliva samples collected during the experiment on the Smart Road required temporary storage in a cooler on wet ice to prevent degradation.

Plasma was separated by refrigerated centrifugation (2000 RPMs for 15 min), aliquoted into cryogenic vials, and stored at −20 °C. Plasma melatonin levels were measured by Surrey Assays Ltd. by direct radioimmunoassay [32].

Saliva specimens were centrifuged at 3000 RPM for 2 min and then aliquoted into cryogenic vials for storage at −20 ˚C until assay. Salivary melatonin levels were measured by Surrey Assays Ltd. (Guildford, UK) by direct radioimmunoassay [33]

### 4.5. Participants

Twenty-nine participants, aged 18 to 30 years, completed the full set of experimental sessions for the study. Across all experimental groups (Driver, Pedestrian, and Light Trespass) 15 males (Mean (M) = 25.26 years, standard deviation (SD) = 3.63 years) and 14 females (M = 23.61 years, SD = 3.71 years) completed the study. The driver group had 5 males (M = 27.2 years, SD = 3.49 years) and 5 females (M = 26.2 years, SD = 2.59 years). The light trespass group also had 5 males (M = 24.2 years, SD = 3.11 years) and 5 females (M age = 23 years, SD = 2.82 years). The pedestrian group had 5 males (M = 24.4 years, SD = 4.15 years) and 4 females (M = 21.5 years, SD = 4.72 years).

### 4.6. Data Analysis

The melatonin results were analyzed using two statistical tests. Data were considered in a time-based analysis where the melatonin concentration values at each time were assessed and using an AUC method where the total melatonin was summed over the time of the exposure. The “No Light” condition was used as the baseline for comparison to each of the experimental street lighting conditions.

Linear Mixed Modelling (LMM) was used to evaluate the fixed effects of light type on melatonin suppression and time. Kolmogorov–Smirnov tests on the dependent variables showed that they were normally distributed. For all statistical tests the level of significance was set at 0.05. Where relevant, Tukey’s honest significant difference was used for post hoc analyses.

## 5. Conclusions

The results of this paper indicate that for the night-time exposure periods tested at recommended roadway exposure levels, as compared to a no roadway lighting condition, the spectrum of the light source made no impact on salivary melatonin level in healthy subjects. This includes all roadway users at the test exposure periods; (drivers (2 h exposure time: 1 A.M. to 3 A.M.), pedestrians (4 h exposure time: 10 P.M. to 2 A.M.), and those experiencing light trespass (2 h exposure time: 12 A.M. to 2 A.M.).

The results indicate that possible additional research can be undertaken to explore the relationship of light more fully to health at night. Such research might include:

- Using plasma-based measurements to provide a more sensitive measure of melatonin to roadway lighting trespass.
- Studying additional durations and intensities to define the boundaries of impact of the roadway lighting more fully on melatonin regulation.
- Obtaining measurement of other physiological and behavioral parameters to show different impacts of light trespass from roadway lighting on human health and well-being.

## Figures and Tables

**Table 1:** Exposure Conditions based on Experiment Type.

	Start Time	Exposure Time	Duration	Roadway Luminance	Corneal Illuminance	Saliva Sampling Rate
Driver	11 P.M.	1 A.M. to 3 A.M.	2 h	1.0 cd/m^2^	1.4 lux	Every 30 min
Pedestrian	8 P.M.	10 P.M. to 2 A.M.	4 h	1.0 cd/m^2^	10.0 lux	Every 40 min
Light Trespass	10 P.M.	12 A.M. to 2 A.M.	2 h	1.0 cd/m^2^	1.5 lux	Every 30 min

**Table 2:** Calculated corneal alpha-opic lux values for the five roadway luminaires utilized in the study.

Condition	5000 K LED	4000 K LED	3000 K LED	2200 K LED	2100 K HPS
S-cone α opic EDI [lux]	0.77	0.50	0.31	0.14	0.09
M-cone α opic EDI [lux]	1.39	1.30	1.23	1.11	0.99
L-cone α opic EDI [lux]	1.44	1.46	1.48	1.53	1.58
Rhodopic EDI [lux]	1.04	0.84	0.73	0.58	0.35
Melanopic EDI [lux]	0.86	0.65	0.55	0.42	0.22

## Data Availability

The data is not currently publically available.
